# Natural Carbon Isotope Abundance of Plasma Metabolites and Liver Tissue Differs between Diabetic and Non-Diabetic Zucker Diabetic Fatty Rats

**DOI:** 10.1371/journal.pone.0074866

**Published:** 2013-09-23

**Authors:** Jean-Philippe Godin, Alastair B. Ross, Marilyn Cléroux, Etienne Pouteau, Ivan Montoliu, Mireille Moser, Sunil Kochhar

**Affiliations:** Analytical Sciences, Nestlé Research Center, Lausanne, Switzerland; Consiglio Nazionale delle Ricerche, Italy

## Abstract

**Background:**

‘You are what you eat’ is an accurate summary for humans and animals when it comes to carbon isotope abundance. In biological material, natural^13^C/^12^C ratio is subject to minute variations due to diet composition (mainly from ingestion of C_3_ and C_4_ metabolism plants) and to the discrimination between ‘light’ and ‘heavy’ isotopes during biochemical reactions (isotope effects and isotopic fractionation).

**Methodology/Principal Findings:**

Carbon isotopic abundance was measured in ZDF (*fa/+*) and ZDF (*fa/fa*), (lean and obese-diabetic rats respectively) fed the same diet. By analysing plasma metabolites (glucose and non-esterified fatty acids), breath and liver tissue by high-precision isotope ratio mass spectrometry, we demonstrate for the first time statistically distinguishable metabolic carbon isotope abundance between ZDF (*fa/+*) and ZDF (*fa/fa*) rats based on plasma glucose, palmitic, oleic, linoleic, arachidonic acids and bulk analysis of liver tissue (P<0.005) resulting into clear isotopic fingerprints using principal component analysis. We studied the variation of isotopic abundance between both groups for each metabolite and through the metabolic pathways using the precursor/product approach. We confirmed that lipids were depleted in ^13^C compared to glucose in both genotypes. We found that isotopic abundance of linoleic acid (C18: 2n-6), even though both groups had the same feed, differed significantly between both groups. The likely reason for these changes between ZDF (*fa/+*) and ZDF (*fa/fa*) are metabolic dysregulation associated with various routing and fluxes of metabolites.

**Conclusion/Significance:**

This work provides evidence that measurement of natural abundance isotope ratio of both bulk tissue and individual metabolites can provide meaningful information about metabolic changes either associated to phenotype or to genetic effects; irrespective of concentration. In the future measuring the natural abundance δ^13^C of key metabolites could be used as endpoints for studying in vivo metabolism, especially with regards to metabolic dysregulation, and development and progression of metabolic diseases.

## Introduction

In medicine and biology, metabolite concentration or metabolic profiling is often used to diagnose disease state and provide insights into metabolic processes [1]. In the diagnosis of diabetes mellitus type 2 (T2DM), fasting plasma or blood glucose concentrations have long been the first indicator of the development of the disease. Recently, other biomarkers such as acylcarnitines and amino acids have also been suggested for the diagnosis of T2DM [2]. One of the goals of biomarker discovery is to detect the onset and development of diseases such as T2DM before they result in biochemically significant changes in systemic biochemistry. Present screening biomarkers for T2DM generally rely on measuring biomarker concentrations, which occurs relatively late in the cascade of the development of the disease. One different approach to disease diagnosis is to measure fluxes through relevant pathways using stable isotopes. Thus, phenotype (defined by the interaction of the gene, nutrients and environment) and its perturbation (e.g. fasting, postprandial and disease conditions) can be more accurately described through the flux measurements of pathways than measuring concentrations alone [3–5].

In the field of ecology, the measurement of the natural ratio of heavy to light atomic isotopes (e.g.^13^C/^12^C^15^, N/^14^N, ^2^H _2_/H_2_ and^18^O/^16^O) in mammalian tissues is a powerful tool to study trophic ecology and energy pathways [6–8]. In chemical archaeology, it has been demonstrated that the isotopic composition of the diet (as^15^N/^14^N from protein and ^13^C/^12^C from protein, carbohydrates and lipids) is reflected in animal or human tissues with some additional *in situ* isotopic discrimination [9]. This in situ isotopic discrimination (isotopic effect and isotopic fractionation) is due to either some enzymes having a slightly different affinity and reaction rate for heavier molecules (enriched in heavy isotopes) compared to normal or lighter weight molecules (fewer heavy isotopes) [10]. Over time, this slight difference in metabolism of heavier molecules can lead to a measurable difference in isotope ratio. The most widespread example of this difference in ‘isotopic fractionation’ is the carbon isotopic abundance of plants with C_3_ and C_4_ photosynthetic pathways [11] (average natural abundance of ^13^C in all matter is about 1.1%, 1.081% in C_3_ plants (e.g. soybean) and 1.0975% in C_4_ plants (e.g. corn). Differences in isotopic enrichment of mammals is not solely dictated by diet (between C_3_-C_4_ or between marine and terrestrial diets), and studies looking at changes due to dietary differences in isotopic composition find that different tissues and animals incorporate the isotopic shift at various rates [12]. Isotopic turnover can vary from few days to several months within a single species depending on many physiological parameters including protein turnover and animal growth [13,14]. Other studies in humans have demonstrated that nitrogen isotope abundance of tissues samples (e.g. hair and liver) can also reflect nutritional stress or eating disorders such as anorexia nervosa and bulimia [15,16]. This relationship is explained by the variation of nitrogen isotope abundance δ^15^N during negative nitrogen balance (associated with isotopic fractionation of nitrogen during deamination and transamination reactions) [17]. Other explorative studies showed that slight natural variation of isotopic abundance of deuterium and ^18^O can have biological meaning in human and mice [18,19].

The isotopic data generated in such studies are generally measured ‘in bulk’ in tissues, plasma or macromolecules (such as lipids and proteins) using an elemental analyzer coupled to isotope ratio mass spectrometer (EA-IRMS) for ^13^C and ^15^N isotopes. However, in plants, changes to natural isotopic abundance have been studied at the intramolecular level [20]. This type of approach is yet to be applied to studies on mammalian metabolism. Several studies have highlighted that “isotopic routing” between various tissues can make the interpretation of isotopic data in mammals a challenging task [21].

**Figure 1:**
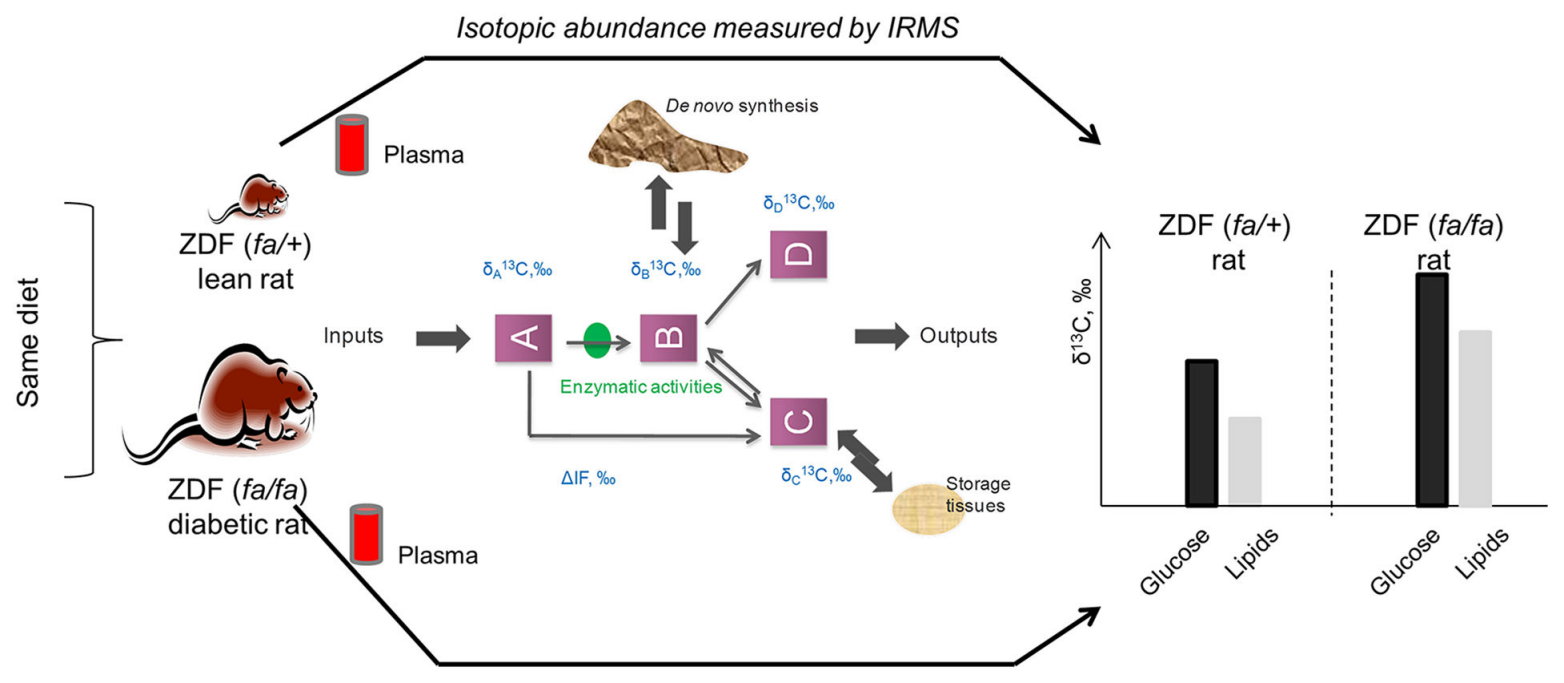
Synopsys of the exploratory study studying the relative impact of the pathophysiological conditions (ZDF (*fa/+*) and ZDF (*fa/fa*) rats) on the natural carbon isotopic abundance (δ^13^C) for individual metabolites measured in plasma, in breath and liver tissue samples by isotope ratio mass spectrometry.

We present here an exploratory study on the relative impact of a pathophysiological condition (diabetics) on the natural carbon isotopic abundance (δ^13^C) for individual metabolites measured in plasma, in breath and liver tissue samples (total liver isotope ratio) from Zucker Diabetic Fatty (ZDF) rats. To our knowledge, there is little published information on how intrinsic physiological and biochemical factors (as opposed to external factors such as diet) can affect natural isotopic abundance. Knowing that the variability of *in vivo* isotopic signatures is an index of combined effects of the diet (e.g. natural enrichment via ingested carbon sources), and other inherent variability related to physiology, we compared the natural abundance isotope ratio of rats that spontaneously develop diabetes (ZDF-*Lepr fa*/Crl; ZDF (*fa/fa*)) and their non-diabetic littermates (ZDF *fa/+*) fed the same diet (Figure **1**). Because of the dietary control, any isotopic difference observed results from expression of the recessive leptin resistance trait (and other possible associated mutations) and the consequent development of diabetes through overeating. If differences in natural isotopic abundance due to metabolic disorders can be detected, this may ultimately shed light on the relevant dysregulated pathways and specific isotopic signatures signalling the presence or onset of metabolic dysregulation.

## Results

### Physiological characteristics and changes to circulating metabolites

ZDF (*fa/fa*) develop diabetes due to being homozygous for a spontaneous mutation that leads to a non-functional leptin receptor as well as a second mutation that impairs β-cell function [22]. The rats are leptin resistant, obese and develop hyperlipidemia, insulin resistance and hyperglycemia in a pattern similar to T2DM [23,24]. Their heterozygous (ZDF, *fa/+*) siblings are non-obese and are phenotypically lean and normoglycaemic. Both groups received the same diets from weaning (commercial rodent feed). Although food intake is more than 1.5 times higher for ZDF (*fa/fa*) rats compared to ZDF (*fa/+*) rats [25], this is not known to influence the isotopic abundance of plasma metabolites or body tissues under these conditions.

As expected, at fifteen weeks of age, ZDF (*fa/fa*) rats had significantly higher body, liver and adipose tissue weights (P<0.01) than ZDF (*fa/+*) rats (Table **1**). Basal plasma level of glucose, triglycerides and free fatty acids were all also significantly higher in ZDF (*fa/fa*) than in ZDF (*fa/+*) rats (P=0.002). Plasma fatty acid composition also differed, with arachidonic (C20: 4n-6) and stearic acid (C18: 0) concentrations at 8.4% and 2.6% lower in ZDF (*fa/fa*) rats compared to ZDF (*fa/+*) respectively, whereas oleic acid (C18: 1) increased by 7.6% (Table **2**). In both genotypes, the most abundant fatty acid was palmitic acid (C16: 0), 24.9 and 30.3% for ZDF (*fa/+*) and ZDF (*fa/fa*) rats respectively.

**Table 1:** Physiological characteristics of ZDF (*fa/+*) and ZDF (*fa/fa*) groups studied at 16 weeks old.

		ZDF (*fa/+*) rat	ZDF (*fa/fa*) rat	P value
**Plasma**	Glucose (mmol/L)	3.31 ± 0.19	7.25 ± 0.81	0.002
	Lactate (mmol/L)	4.06 ± 0.65	7.82 ± 2.43	0.009
	Triglycerides (mmol/L)	0.3 ± 0.07	2.62 ± 0.35	0.002
	Palmitic acid (mmol/L)	2.67 ± 0.06	7.31 ± 0.59	0.002
	Stearic acid (mmol/L)	1.42 ± 0.03	2.62 ± 0.09	0.002
	Oleic acid (mmol/L)	0.78 ± 0.08	3.66 ± 0.34	0.002
	Linoleic acid (mmol/L)	2.41 ± 0.07	5.22 ± 0.39	0.002
	Arachidonic acid (mmol/L)	3.39 ± 0.12	5.72 ± 0.35	0.002
**Tissues**	Body weight (g)	283 ± 8	327 ± 14	0.002
	Liver weight (g)	6.6 ± 0.07	14.4 ± 0.6	0.002
	Adipose weight (g)	1.33 ± 0.07	4.89 ± 0.29	0.002

The data are expressed as median ± SEM.

**Table 2:** Plasma fatty acid composition.

Fatty Acid	ZDF (*fa/+*) rat	ZDF (*fa/fa*) rat	P value
Palmitic acid, %	24.95 ± 0.34	30.35 ± 0.32	< 0.001
Stearic acid, %	13.19 ± 0.20	10.83 ± 0.26	< 0.001
Oleic acid, %	7.03 ± 0.43	15.21 ± 0.41	< 0.001
Linoleic acid, %	22.0 ± 0.40	22.84 ± 0.38	0.086
Arachidonic acid, %	32.16 ± 0.91	21.91 ± 0.67	< 0.001

All values are expressed as mol % ± SEM. Fatty acid names: C16 (palmitic acid), C18 (stearic acid), C18: 1 (oleic acid), C18: 2 (linoleic acid), and C20: 4 (arachidonic acid).

### Variation in isotopic abundance between ZDF (*fa/+*) and ZDF (*fa/fa*) rats

Measurement of isotopic abundance of plasma palmitic, stearic, oleic, linoleic and arachidonic acids, glucose, breath carbon dioxide (CO_2_) and liver tissue from both groups of rats were carried out using high-precision isotope ratio mass spectrometry hyphenated to different peripherals [26,27] (IRMS) (see Materials and Methods).

Isotopic abundance (δ^13^C) was not significantly different between ZDF (*fa/+*) and ZDF (*fa/fa*) for breath CO_2_, whereas liver tissue was significantly different (P=0.004) (Table **3**). The difference of carbon isotope abundance for liver tissue (Δ^13^C) between ZDF (*fa/fa*) and ZDF (*fa/+*) was 5.06‰, and was the largest natural isotopic difference observed in this study. Similar observations were reported for specific compounds measured in plasma: glucose, palmitic, oleic, linoleic and arichidonic acids had significantly different δ^13^C (P<0.005) with Δ^13^C calculated at 2.91, 3.30, 2.45, 3.07 and 1.56‰ respectively. The observed range of isotopic variation indicated that the isotope abundance was always greater (enriched in ^13^C atoms) in ZDF (*fa/fa*) rats compared to lean rats. One exception was stearic acid, which did not differ in isotopic abundance between both genotypes (P > 0.2).

**Table 3:** Carbon isotopic abundance (δ^13^C, ‰) metabolites, breath and liver samples measured by isotope ratio mass spectrometer hyphenated to various peripherals for ZDF (***fa/***+)** and ZDF (*fa/fa*) rats.**

	ZDF (*fa/*+) rat	ZDF (*fa/fa*) rat	Δ ^13^C, ‰	P value
	δ^13^C, ‰	δ^13^C, ‰		
Breath CO_2_	-23.24 ± 0.35	-22.73 ± 0.11	0.59	0.266
Glucose	-25.23 ± 0.87	-22.32 ± 0.15	2.91	0.002
Palmitic acid	-27.37 ± 1.61	-23.91 ± 0.25	3.30	0.004
Stearic acid	-26.76 ± 1.51	-24.63 ± 0.42	1.80	0.394
Oleic acid	-25.58 ± 1.01	-22.91 ± 0.11	2.45	0.004
Linoleic acid	-29.52 ± 1.08	-26.53 ± 0.22	3.07	0.002
Arachidonic acid	-28.13 ± 0.38	-26.58 ± 0.18	1.56	0.004
Liver tissue	-21.43 ± 0.31	-16.37 ± 0.53	5.06	0.004

Δ^13^C is the isotopic difference for each metabolite between ZDF (*fa/*+ and ZDF (*fa/fa*) rats.

### Isotopic fractionation within each genotype

The biochemical relationships between glucose and fatty acids or between each fatty acid are not straightforward within an *in vivo* model due to various metabolic fluxes of fatty acids and glucose between organs and the existence of many intermediates or branching points. However, it is possible to gain additional information by considering the precursor/product relationship between two metabolites through the calculation of absolute isotopic difference (in ‰) as an index of isotopic fractionation. The isotopic fractionation between plasma glucose and palmitic acid for ZDF (*fa/fa*) and ZDF (*fa/+*) was calculated at 2.01 and 2.39‰ respectively (P=0.7) (Table **4**) indicating that no isotopic perturbation of this pathway was observed in these conditions. Similarly, no statistical differences between the two genotypes were measured for biochemical reactions between stearic and oleic acids and between polyunsaturated fatty acids (linoleic to arichidonic acids). Conversely, for the conversion of palmitic to stearic acid, there was a significant difference in isotopic fractionation between the two genotypes (P=0.007). This difference between both groups (for ZDF (*fa/+*) and ZDF (*fa/fa*) rats) in isotopic abundance for palmitic to stearic acids (δ^13^C_palmitic_ -δ^13^C_stearic_) was -1.04 ± 0.40‰ and 0.76 ± 0.19‰ respectively.

**Table 4:** Isotopic fractionation (ΔIF, in ‰) measured for key metabolic transitions between a precursor and its product in lean (ZDF, (*fa/+*)) and diabetic (ZDF (*fa/fa*)) rats.

Precursor/product	ZDF (*fa/+*) rat	ZDF (*fa/fa*) rat		P value
	ΔIF,‰	ΔIF,‰		
Glucose/C16:0	2.39 ± 0.38	2.01 ± 0.36		0.877
C16:0/C18:0	-1.04 ± 0.4	-0.76 ± 0.18		0.007
C18:0/C18:1	- 0.04 ± 0.64^#^	-1.67 ± 0.28		0.103
C18:2/C20:4	-1.31 ± 0.45	- 0.006 ± 0.53^#^		0.232

ΔIF, ‰ (δ_A_ - δ_B_) where A/B is the precursor/product ratio. Based on the Wilcoxon Signed Rank Test, all the data were significantly different from zero except for those marked with “#”.

## Discussion

### Potential sources of isotopic changes between genotype

The present data suggest that natural isotopic abundance at the molecular level could be a new approach to obtain signatures and information about the onset of metabolic dysregulation. In contrast to other studies on the effect of past diet on tissues (e.g. bone and hair), we have detected differences that are not due to diet, with a different disease state appearing to be responsible for the difference in observed *in vivo* molecular isotopic abundance. As the system is pushed out of balance (loss of homeostasis), a dysregulation of the storage and transport of many nutrients such as glucose, lipids and amino acids occur. Although the cause-effect relationship behind the difference in δ^13^C signature is complex when multiple compartments are modelled [9], we can then assume that isotope discrimination can occur at various steps between the plasma and the different tissues, including 1) isotope fractionation associated with the uptake and utilization of substrate (such as the gluconeogenic precursors); 2) isotopic fractionation associated during elongation and desaturation of fatty acids, fatty acid oxidation and cellular transport between cytoplasma and mitochondria; 3) isotopic fractionation associated to fatty acid mobilization from adipose tissue; 4) isotopic fractionation associated to *de novo* synthesis; 5) isotopic fractionation associated to changes in the feed composition intake (i.e. the amount of macronutrients ingested) leading to further modulation of enzymatic activity through changes in substrate concentration.

### ^13^C isotopic changes between genotype

Using an *in vitro* model, it was established that the natural isotopic abundance of carbon is not uniformly distributed among the various classes of metabolites [28]. For example, lipids are generally depleted in ^13^C relative to carbohydrates. This isotope fractionation effect occurs during the decarboxylation of pyruvate by pyruvate deshydrogenase, resulting in depleted acetyl-CoA at the carbon 2 position. The low levels of enrichment at natural abundance mean that only total ^13^C-enrichment, and not the intramolecular ^13^C distribution, can be determined. However, we confirmed that the lipids measured were depleted in δ^13^C compared to glucose in both genotypes. This result indicates a consistent relationship in the isotopic fractionation at the branching points for the acetyl-CoA used for both genotypes.

**Figure 2:**
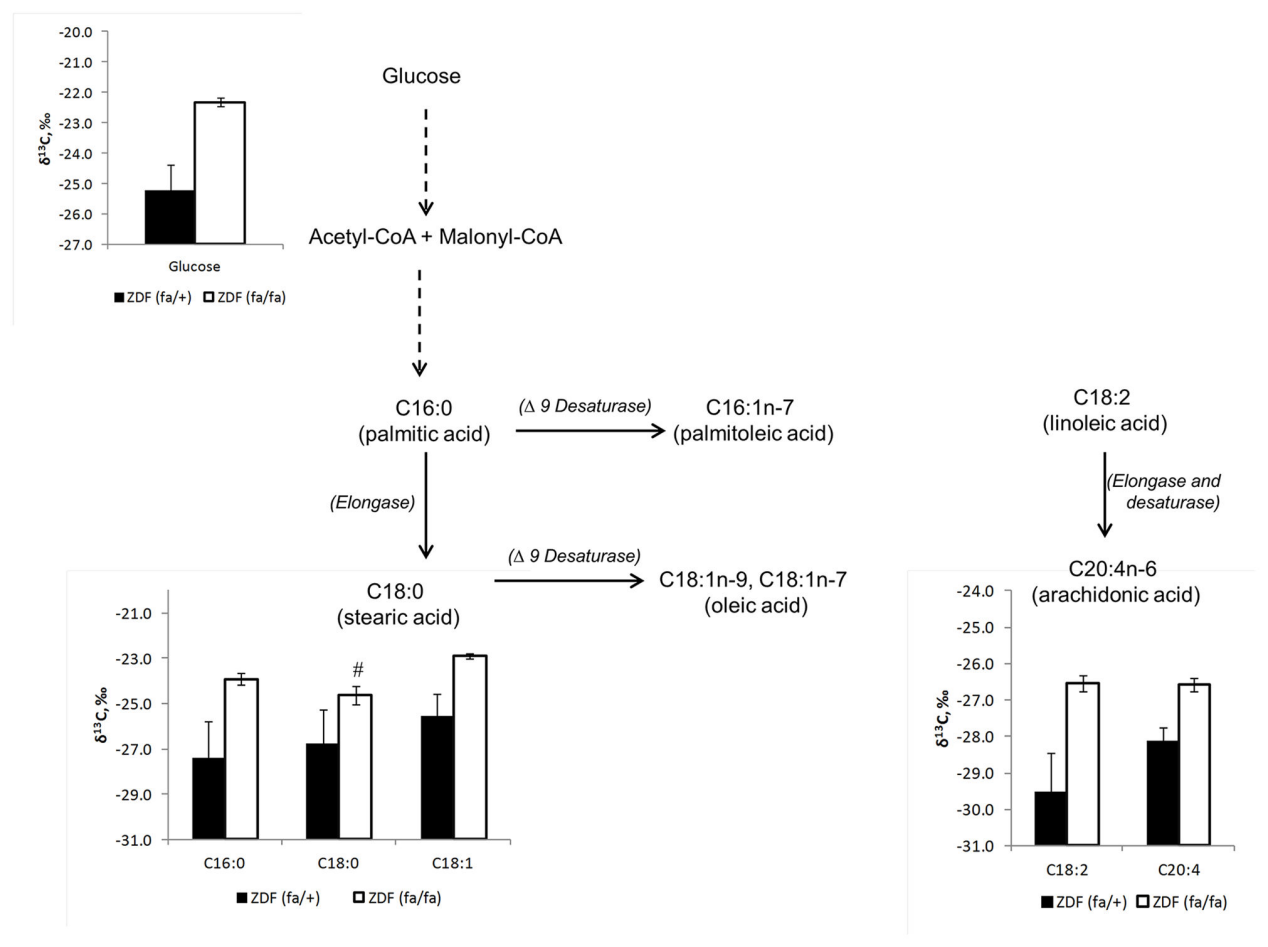
Schematic representation of the variation of carbon isotopic abundance (expressed in δ^13^C, ‰) of plasma glucose, plasma lipids in ZDF (*fa/+*) and ZDF (*fa/fa*) rats. All the data were significantly different between both groups except for stearic acid (indicated by ‘#’).

All plasma metabolites were more enriched in ^13^C (Δ^13^C) in ZDF (*fa/fa*) rats (P < 0.005) except for stearic acid (Figure **2**). A previous study on dogs found that that carbon 1 in plasma glucose was slightly enriched in diabetic fasting dogs compared to healthy dogs (δ^13^C= -23.2 ± 2.3‰ vs. -17.8± 2.1‰ for normal versus diabetic dogs) [29], a similar pattern to that observed for total glucose in this study. The cause-effect relationship for the greater isotopic abundance of plasma glucose in the ZDF (*fa/fa*) rats is difficult to demonstrate. However, it is possible to surmise that the source and routing of glucose (i.e. incomplete suppression of hepatic glucose production and a decreased efficiency of the liver and muscle glucose uptake) differing between both groups may lead to changes in glucose concentration which also impact the concentration of light and heavy isotope. But a clear link between glucose transports, various uptake- of glucose and isotopic differences need to be clarified.

In our study, the fatty acid composition of the plasma of the ZDF (*fa/fa*) rats was distinctly different from the plasma of the ZDF (*fa/+*) with higher % of palmitic and oleic acids whereas the percentage of stearic and arachidonic acids were higher for ZDF (*fa/+*). Isotopically, we found that palmitic, oleic, linoleic and arachidonic were significantly different between ZDF (*fa/fa*) and ZDF (*fa/+*) with the exception of stearic acid. These data suggest that the source of such fatty acids is different between both groups. Assuming that plasma fatty acids are a mixture of newly synthesized fatty acids (*de novo* lipogenesis), products of lipolysis and dietary lipids, the significant isotopic results at natural abundance between both groups can be explained by the rate of *de novo* synthesis and the rate of lipolysis and the influx of dietary lipids. In several studies, when leptin and/or leptin receptor are unregulated, as in the ZDF (*fa/fa*) rat, lipogenesis is also unregulated, leading to an increase of the percentage of *de novo* synthesis for palmitate (around 20%), stearate (around 26%) and oleate (around 44%) between ZDF and Zucker lean fed with a low fat diet [30]. Circulating fatty acids also come from lipolysis of pre-existing adipose stores. Therefore, we cannot exclude that this was an important source of fatty acids in our study that could explain the difference in δ^13^C. It has been reported in ^13^C-tracer studies that the isotope ratio of plasma stearate may be altered due to variation in membrane phospholipase activity and circulating phospholipids, as well as stearate derived from lipolysis occurring in adipose tissue [31].

Linoleic acid (C18: 2n6) is an essential polyunsaturated fatty acid (PUFA) and the starting substrate for n6 PUFA metabolism. Therefore it would be expected that linoleic acid would mainly be derived from dietary sources. Surprisingly, in our study, the Δ^13^C between both genotypes was measured at 3.07‰ (P=0.0004), suggesting that its source was not similar between both groups. Arachidonic acid, which is further downstream in the n6 pathway, was also isotopically different between both genotypes (P=0.002). Rhee et al. observed that linoleic acid isotopic abundance in human healthy plasma behaved differently from the other circulating fatty acids with a lower isotopic ratio (about 2‰) compared to the same dietary fatty acid [32]. Although direct evidence remains to be demonstrated, we interpret this result as indicating an increased production and utilization of this essential fatty acid in both genotypes, though we cannot exclude a small contribution of *de novo* synthesis of C18: 2n6 from other lipids such as C16: 2n6 [33].

Theoretically, under steady state conditions there should be no difference in the precursor/product relationship in a metabolic pathway, even if pool sizes or fluxes differ, unless there is isotopic fractionation specific for that precursor to product reaction [34]. This theoretical consideration appears to hold for the precursor/product ratio of fatty acid synthesis measured in this study (Table 4). Of the ratios measured, only the conversion of palmitic to stearic acid differed (P=0.007) between ZDF (*fa/fa*) and ZDF (*fa/+*) rats. This is in spite of around 86-91% of plasma stearate being derived from palmitate elongation in both Zucker lean and ZDF rats fed on low fat diet [35]. Further studies are needed to decipher the cause-effect relationship of isotopic abundance of fatty acids based on measuring information at the level of individual carbon atoms requiring the development of natural abundance intramolecular isotope measurements [36].

### Isotopic signature of ZDF (*fa/fa*) versus ZDF (*fa/+*) rats

**Figure 3:**
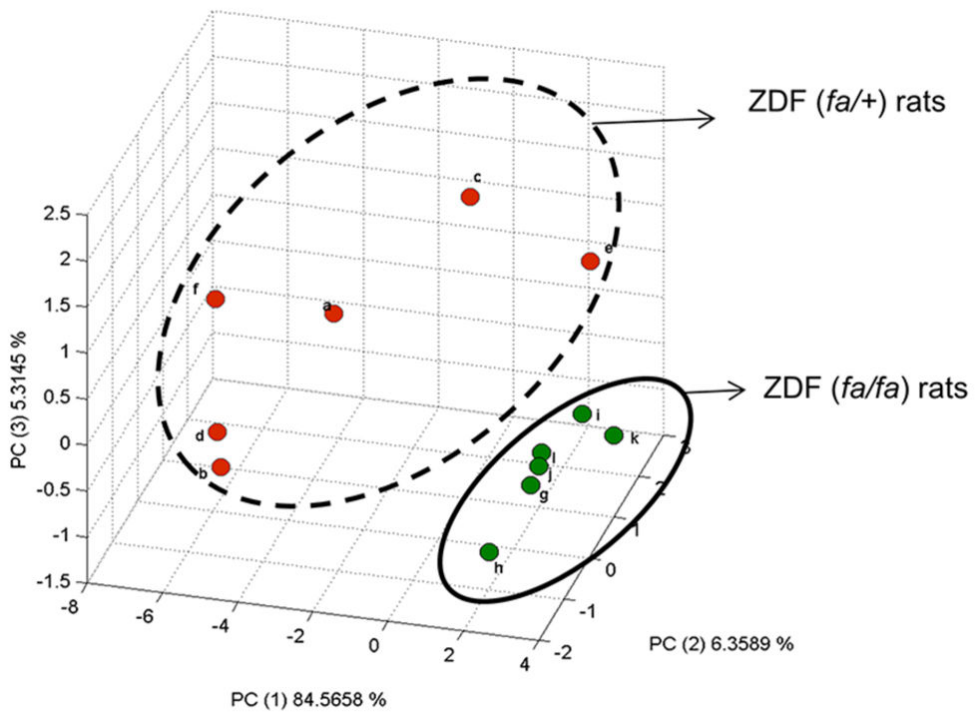
Principal component analysis (PCA) score plots of isotopic abundance of plasma metabolites measured by isotope ratio mass spectrometers (i.e. glucose, palmitic, oleic, stearic, linolenic and arachidonic acids) between ZDF (*fa/+*) and ZDF (*fa/fa*) rats. Red dots are for ZDF (*fa/+*) whereas green ones stand for ZDF (*fa/fa*) rats. Letters are the identification code of the rat. Based on the PCA, there is a clear separation of both groups along PC1 and PC3, confirming that there is an isotopic signature at natural abundance that separates ZDF (*fa/+*) and ZDF (*fa/fa*) rats.

Further analysis using principal component analysis (PCA), based on carbon isotopic abundance of plasma metabolites, showed that ZDF (*fa/fa*) and ZDF (*fa/+*) rats were separated along the first component (Figure **3**). The separation of both genotypes is explained by all the plasma metabolites measured, with glucose, palmitic acid, oleic acid and linoleic acid having the most influence on the model. The separation of the two groups along that the first component suggests that the δ^13^C measurements of these metabolites could serve as an isotopic fingerprint or signature to distinguish these two groups, though if this difference is due to metabolic or direct genetic effects remains to be determined. Of note, δ^13^C variation was greater for ZDF (*fa/+*) rats compared to ZDF (*fa/fa*) rats for all the metabolites measured with the reverse observed for the metabolites concentrations. As the group size used in this study is low, further works with larger populations are needed before conclusions can be made about the importance of intra and inter-group variability for δ^13^C values.

### Metabolic flexibility

The ability to adaptively partition metabolic fuel among various tissues has crucial impact on health and diseases [37]. This concept is often referred to as metabolic flexibility, and is in part based on the ability of skeletal muscle to switch from lipid to carbohydrate oxidation under various conditions and is closely associated with the development of insulin resistance. For example, obesity and T2D are associated with an impaired fat oxidation during the fasting state [38]. Although no changes were found in breath ^13^CO_2_, the slightly higher carbon isotopic abundance in metabolites related to energy metabolism in diabetic rats may be an indicator that pathways to metabolise energy substrates has been compromised, possibly by an increased flux through these pathways for an extended period of time. We speculate here that the isotopic information gathered in both physiological conditions can represent a proxy of metabolic flexibility characterizing the two groups studied. However, further studies are needed to assess if impaired fat oxidation may have an impact on the isotopic signature of oxidation that may correlate with the natural abundance isotopic pattern of plasma metabolites.

### Further work

Although isotopic variation in plasma metabolites confirmed the existence of natural abundance signatures between ZDF (*fa/+*) and ZDF (*fa/fa*) rats, additional isotopic measurements of specific components in the diets and in metabolically important tissues such as liver, pancreas and adipose tissue are needed. Mass balance modelling could then be used to shed further light on whole body mechanisms behind changes to natural abundance of individual metabolites.

In conclusion, we showed distinct natural abundance ^13^C fingerprints between ZDF (*fa/+*) and ZDF (*fa/fa*) rats for individual metabolites in plasma and liver tissue. This difference may result from the exchange of fluxes of metabolites between organs, and/or changing of pathways for *de novo synthesis* associated to isotopic fractionation between light and heavy isotopes. The use of isotopic fingerprints associated with metabolic modelling could lead to the isotopic abundance of key metabolites being a new dimension in understanding the genesis and progression of metabolic diseases. Further studies are also needed to determine if the changes in carbon isotopic abundance occurs before there is a difference in concentration to know if isotope fingerprinting could be a potential approach for early stage diagnostics.

## Materials and Methods

### Animal study

The pre-clinical study was carried out under the Swiss national guidelines and the protocol approved by the local Cantonal Veterinary Office of Vaud, Switzerland. ZDF (*fa/fa*) (diabetic) and (*fa/+*) (lean controls) rats (Charles River SA, France) were used in this study. Both groups of rats (n=6) were fed the same diet from weaning (Purina 5008 (St Louis, MO) from weeks 4-13 and Kliba Nafag 3434 (Provimi Kliba AG, Kaiseraugst, Switzerland) for weeks 13-15). Both diets are grain-based and formulated to be a complete rodent diet. Access to standard pelletized diets and water were ad libitum. At age 16 weeks, rats were weighed and then sacrificed under isofluorane anaesthesia and tissues (liver) were collected, weighed, frozen in liquid nitrogen and then stored at -80°C until analysis. Blood was collected from the carotid artery into Li-heparin tubes and plasma obtained after centrifugation at 1000 g for 10 min. Plasma samples were snap frozen in liquid nitrogen and stored at -80°C before analysis. Breath samples were collected by placing rats in a glass chamber and collecting expired air via a syringe after 5 min. The syringe was equilibrated with two volumes, before a third sample was collected and injected into an expired air analysis vial and analysed within one week of sampling.

### Stable isotope notation

The delta notation is defined as follows: δ^13^C sample, ‰ = (Rs / R_ref_ -1) × 1000, where Rs is the ratio of^13^C/^12^C in the sample and R_ref_ is the^13^C/^12^C ratio of the international standard used (Vienna PeeDee Belemnite).

### Analyses and analytical methods

Breath ^13^CO_2_ analysis was carried out using a GC-IRMS (Delta V, Advantage high-precision IRMS Thermo, Bremen, Germany) using a Poraplot GC column heated to 50°C. The GC-IRMS system, used to measure the breath CO_2_, was calibrated using three reference gases (Eurisotop, France) with certified values: δ^13^C at -22.8, -13.2 and -7.3‰. Typical precision (1σ) of breath isotopic analysis was 0.15‰.

Glucose concentration was measured using a commercial kit (Roche, Basel, Switzerland). Glucose isotopic analysis was carried out on a MAT252 IRMS (Finnigan MAT, Bremen, Germany) coupled to an LC Isolink® interface (Thermo Electron, Bremen, Germany). The LC-IRMS system was calibrated using two international reference materials IAEA-CH6 and USGS-40, with δ^13^C at -10.6 and -28.69‰ respectively. Typical precision (1σ) based on inter-day repeatability of a standard glucose sample at natural abundance was 0.4‰ [39].

Liver isotopic analyses (performed on approximately 100 mg of wet tissue) were carried out with a flash elemental analyzer (EA) coupled to a Delta V advantage high-precision IRMS (Thermo, Bremen, Germany). The liver tissue was not homogenised.

Lipid analyses (concentration and isotopic analysis) were carried out with a gas chromatography-combustion-isotope ratio mass spectrometer (GC-C-IRMS) (Thermo, Bremen, Germany). Briefly, a gas chromatograph (GC) was connected to an 850°C combustion furnace coupled to a Delta V advantage high-precision IRMS. The GC carrier gas was helium and was diverted to IRMS and FID devices with a split ratio of 1/10. Fatty acids were transesterified to their methyl esters before analysis [40]. The GC-C-IRMS system was calibrated using a mix of three methylated fatty acids (C15: 0 with a δ^13^C at -30.22‰; C20: 0 with a δ^13^C at -33.06‰ and C25: 0 with a δ^13^C at -28.21‰) (Chiron A/S, Trondheim, Norway). In each sample, an internal standard (i.e. C23: 0 as FAME) was spiked. Typical precision (1σ) based on inter-day repeatability on plasma samples was 0.6‰ (n=57). Fatty acid isotopic analyses were carried out in splitless mode on a DB-23 capillary column (60m x 0.25mm x 0.25µm film thickness, J&W Scientific, Folsom, USA). The GC oven was programmed as follows: 50°C held for 1 min, increased to 175°C at 25 °C/min, increased to 210°C at 10 °C/min and finally increased until 235°C at 5 °C/min and held 8 min.

### Statistical analyses

Data were not normally distributed so median and standard error of the median (based on the robust standard deviation Sn of Rousseeuw) are used throughout. The Wilcoxon rank sum test was used to determine whether groups were different and the Hodges-Lehmann estimator used to quantify the median difference between the two groups. Univariate analysis was performed with R 2.6.1 (http://www.R-project.org).

Data pre-treatment, correlation analysis and principal component analysis (PCA) were performed on Matlab™ 7.9 (The Mathworks, Inc., MA, USA). In-house routines were used for importing data and visualization, while pre-processing and PCA modelling were done using the PLS-Toolbox v 5.8 (Eigenvector Research Inc., WA, USA). Coefficients of determination (r^2^) were calculated using Excel (Microsoft Corp., Redmond, WA).
